# First report of Usutu virus fatal infections in Chilean tinamous (*Nothoprocta perdicaria*), brahminy starlings (*Sturnia pagodarum*), and multiple other bird species in zoological gardens and wildlife in the Czech Republic

**DOI:** 10.1186/s42522-025-00191-3

**Published:** 2026-01-02

**Authors:** Jan Kamiš, Veronika Grymová, Petr Suvorov, Luc Tardy, Petr Vrána, Jan Kirner, Soňa Peková, Vladimír Piaček, Miša Škorič, Jan Pokorný, Natalie Rudenko, Martin Palus, Václav Hönig

**Affiliations:** 1 Laboratory of Arbovirology, Institute of Parasitology, Biology Centre of the Czech Academy of Sciences, Branišovská 31, České Budějovice, CZ-37005 Czechia; 2 Faculty of Science, University of South Bohemia, Branišovská 31, České Budějovice, CZ-37005 Czechia; 3 Avetum Veterinary Clinics, Tumaňanova 11/9, Brno-Mokrá Hora, CZ-62100 Czechia; 4 Zoo Brno and Environmental Educational Centre, U Zoologicke zahrady 46, Brno, CZ-63500 Czechia; 5 Zoological Garden Ostrava, Michalkovická 2081/197, Ostrava, CZ-71000 Czechia; 6 Zoological Garden Olomouc, Darwinova 29, Olomouc, CZ-77900 Czechia; 7 Tilia Laboratories, 5. května 44, Pchery, CZ-27308 Czechia; 8 Faculty of Veterinary Hygiene and Ecology, University of Veterinary Sciences Brno, Palackého tř. 1946/1, Brno, CZ-61242 Czechia; 9 Faculty of Veterinary Medicine, University of Veterinary Sciences Brno, Palackého tř. 1946/1, Brno, CZ-61242 Czechia; 10 Zoological and Botanical Garden Pilsen, Pod Vinicemi 928/9, Plzen, CZ-30100 Czechia; 11 Laboratory of Molecular Ecology of Vectors and Pathogens, Institute of Parasitology, Biology Centre of the Czech Academy of Sciences, Branišovská 31, České Budějovice, CZ-37005 Czechia; 12 Laboratory of Emerging Viral Infections, Veterinary Research Institute, Hudcova 296, Brno, CZ-62100 Czechia; 13 Department of Experimental Biology, Faculty of Science, Masaryk University, Kamenice 735, Brno, CZ-62500 Czechia; 14 National Institute of Public Health, Šrobárova 49, Praha, CZ-10000 Czechia

**Keywords:** Usutu virus, Flavivirus, Chilean tinamou, Brahminy starling, Owls, Blackbirds, Zoological gardens, Serology, Cross-reactivity

## Abstract

**Background:**

The Usutu virus (USUV; *Orthoflavivirus*, *Flaviviridae*) is a mosquito-borne pathogen causing fatal neuroinfections in susceptible wild and captive birds, particularly blackbirds, other passerines, and owls. Zoological gardens provide favourable conditions for the circulation of such viruses due to the proximity of diverse species and limited options for prevention.

**Methods:**

Following the sudden death of several Chilean tinamous kept in the Brno zoological garden, we tested tissues sampled from 22 bird cadavers (from zoos, private owners, and free-living birds) for the presence of USUV and West Nile virus (WNV) RNA using duplex reverse transcription qPCR. Near-complete whole-genome sequences were acquired from positive samples by next-generation sequencing and subjected to phylogenetic analyses. Furthermore, serum samples from additional zoo animals and privately owned birds were screened for anti-flavivirus antibodies using ELISA and subsequently confirmed by the virus neutralization test.

**Results:**

We report fatal USUV infections in multiple bird species from three zoological gardens in the Czech Republic. Duplex RT-qPCR targeting USUV and West Nile virus (WNV) detected USUV RNA in tissues from two Boreal owls (*Aegolius funereus*), one Eurasian pygmy owl (*Glaucidium passerinum*), two Brahminy starlings (*Sturnia pagodarum*), and four Chilean tinamous (*Nothoprocta perdicaria*). Additionally, three randomly found cadavers of free-living blackbirds (*Turdus merula*) tested positive. Pathological findings ranged from minimal pathological changes to pronounced hepatosplenomegaly with intestinal bleeding. Phylogenetic analysis of near-complete genome sequences assigned all viruses to the Europe 2 genetic lineage, revealing partial geographic clustering among isolates obtained in this study. Serological testing confirmed exposure in additional birds and demonstrated cross-neutralisation between anti-USUV and anti-WNV-positive sera.

**Conclusions:**

In zoological gardens, flavivirus infections can cause substantial losses, even among rarely bred or endangered species. Given the zoonotic potential of both USUV and WNV, documenting their occurrence in avian hosts is important not only for animal health but also for human disease surveillance from the One Health perspective.

**Supplementary Information:**

The online version contains supplementary material available at 10.1186/s42522-025-00191-3.

## Background

The Usutu virus (USUV) is a mosquito-borne flavivirus (*Flaviviridae*, *Orthoflavivirus*) first described in 1959 in *Aedes neavei* mosquitoes in southern Africa [1, 2]. The virus was likely introduced to Europe as early as the 1950s, first to Spain and subsequently at least twice more via migratory birds [3, 4]. It is currently present in most western, southern, and central European countries including the Czech Republic, with multiple genetic lineages known to (co-)circulate [1, 5–7]. *Culex pipiens* is considered the principal vector of USUV in Europe [8].

The introduction of USUV to new geographical areas is often associated with high mortality in susceptible wild bird species, particularly blackbirds, other passerines, and owls, as well as in captive birds, including exotic species kept in zoological gardens [9–12]. Fatal flavivirus infections in endangered species or trained falconry birds can result in considerable losses, including financial ones. Prevention options are limited, as no targeted vaccine or treatment is approved for use in birds [13, 14].

Beyond its veterinary impact, USUV is of increasing zoonotic concern. By 2022, 112 acute human infections had been reported in Europe, including 30 cases of neuroinvasive disease [15]. However, the true number of symptomatic cases is likely underestimated, as mild cases may remain undiagnosed or be misdiagnosed as West Nile virus (WNV) or tick-borne encephalitis virus (TBEV) infections, due to serological cross-reactivity among these flaviviruses [5, 16].

Here, we report a series of fatal USUV infections in wild birds and birds housed in zoological gardens in the Czech Republic, including the first cases in Chilean tinamous (*Nothoprocta perdicaria*), a species rarely bred in Europe and in Brahminy starlings (*Sturnia pagodarum*).

## Materials and methods

### Sampled birds

**Fig. 1:**
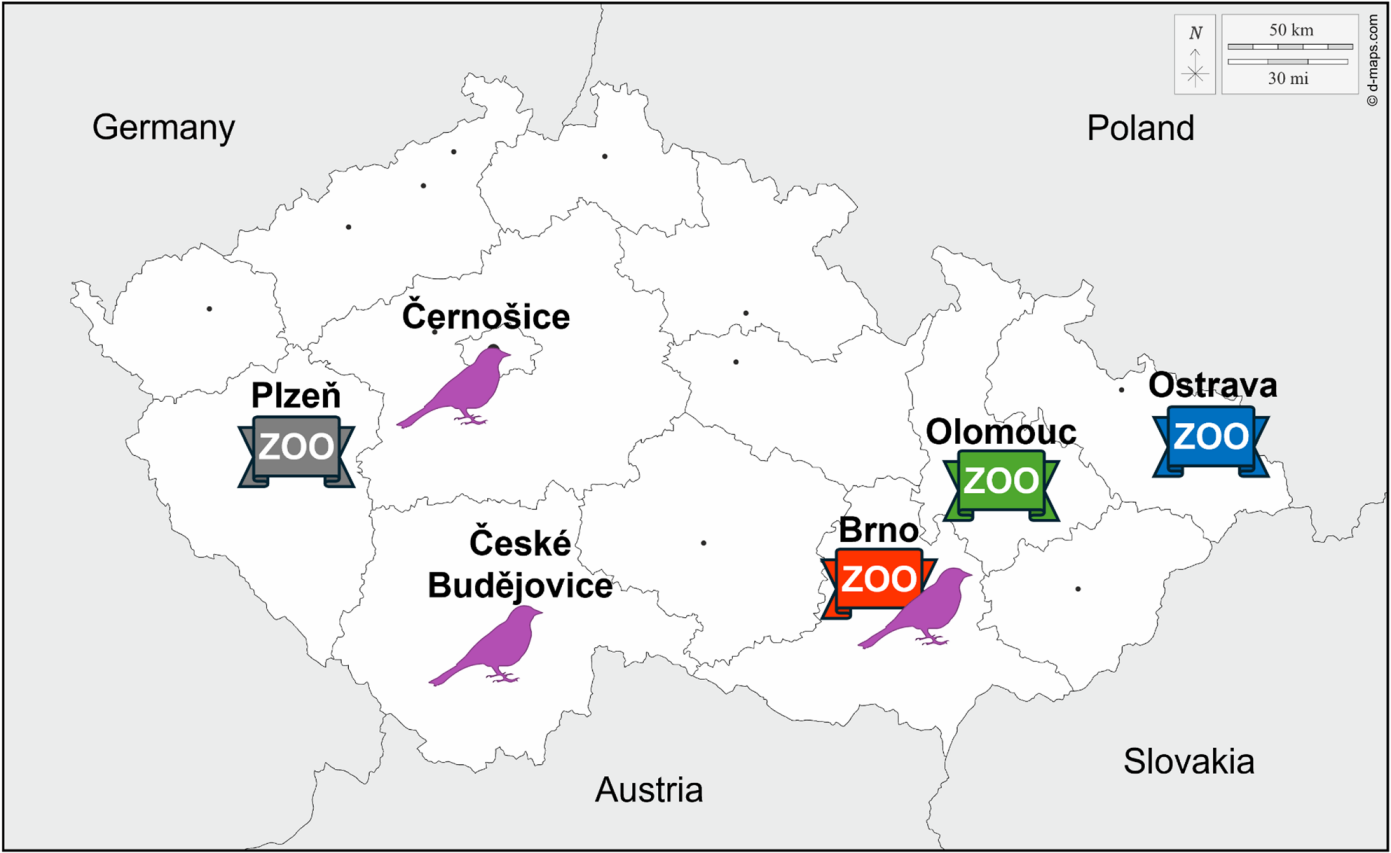
Geographic localization of the zoological gardens in which the sampled birds were kept (grey - Plzeň Zoo, red - Brno Zoo, green - Olomouc Zoo, blue - Ostrava Zoo) and locations of the randomly found cadavers of blackbirds (violet bird icon)

The samples were obtained from randomly found cadavers of wild birds with no other apparent cause of death, or from captive-bred birds (zoological gardens or private owners) between June and September 2024. One additional free-living blackbird was found and sampled in August 2022. The geographic localization of the zoological gardens as well as the locations where the cadavers of free-living birds were found are depicted in Fig. 1. Detailed data of sampled individuals are listed in the Supplementary Table 1.

### Sample processing and screening for USUV and WNV RNA

Bird cadavers were dissected in biohazard boxes under sterile conditions. Each organ was removed using sterile tools to prevent cross-contamination between samples. Collected samples (brain, heart, kidney, liver, lungs, spleen or a mixture of the tissues, when individual organs were unavailable) were homogenized to create 20% (w/v) suspensions in C6/36 cell culture medium (L15, 10% fetal bovine serum, 5% tryptose phosphate broth, 1% l-glutamine, 100 U/mL penicillin, and 100 µg/mL streptomycin) using 5 mm sterile stainless steel beads in Tissue Lyzer II (Qiagen, Hilden, Germany). RNA was isolated from 200 µl of the cleared homogenate using MagMAX Viral/Pathogen Nucleic Acid Isolation Kit (Thermo Fisher Scientific, Waltham, Massachusetts, USA) in KingFisher Apex system (Thermo Fisher Scientific, Waltham, Massachusetts, USA). USUV and WNV RNA were detected in 5 µl of extracted RNA using a duplex one-step reverse transcription quantitative polymerase chain reaction (RT-qPCR) using SuperScript III One-Step RT-PCR System with Platinum™ Taq DNA Polymerase (Invitrogen, Carlsbad, California, USA) and targeting a portion of NS5 coding sequences using published sets of primers and probes [17, 18] (Supplementary Table 2). Synthetic single-stranded standards for each of the detected viruses were included to generate calibration curves which allowed absolute quantification of the targets.

### Next generation sequencing and phylogenetic analyses

From all RT-qPCR-positive individuals, the sample with the lowest Ct value was selected for next generation sequencing. Tiled amplicon sequencing [19], based on a previously developed USUV-specific primer panel [20] was performed on a MinIon Mk1B device (Oxford Nanopore Technologies, Oxford, United Kingdom). Briefly, viral RNA was reverse transcribed using LunaScript RT SuperMix (New England Biolabs, Ipswich, Massachusetts, USA) with random hexamer priming, followed by multiplex PCR in two pools to produce 32 overlapping amplicons of approximately 500 bp each. The amplicons were pooled, cleaned using AMPure XP beads (Beckman Coulter Life Sciences, Indianapolis, Indiana, USA), and 200 fmol used for sequencing library preparation. Samples were barcoded and adapter-ligated according to the Oxford Nanopore Technologies Ligation sequencing amplicons – Native Barcoding SQK-NBD114.24 protocol (NBA_9168_v114_revR_30Jan2025) and run on a R10.4.1 (FLO-MIN114) flow cell (Oxford Nanopore Technologies, Oxford, UK) with a target yield of 20 K pass-reads per barcoded sample. Basecalling and demultiplexing of sequencing data was conducted in MinKNOW 24.11 using the Dorado Super High Accuracy 4.3 basecalling algorithm, quality control, filtering, and assembly in Linux (Ubuntu v. 22.04.3 LTS) using samtools and FastQC, followed by the ARTIC pipeline for trimming, polishing, reference-guided assembly to the USUV reference genome NC_006551.1. Consensus sequences were generated using Geneious Prime v.2025.1.3 (Dotmatics, Boston, Massachusetts, USA). In addition to the newly detected USUV strains, we sequenced three USUV strains (202TM10, 208TM10, and 264TM10) from the brains of infected blackbirds reported previously [7]. The sequences were submitted to the GenBank database under accession numbers PX210786-PX210800. The sequences were aligned with sequences representing different USUV genetic lineages obtained from the GenBank database and truncated to uniform length (10,936 nt). GTR + R substitution model was selected as best suitable using Smart Model Selection tool [21]. Maximum likelihood [22] and Bayesian inference [23] phylogenetic trees were generated in Geneious Prime 2025.1.3.

### Virus isolation in cell culture and immunofluorescent staining of viral antigens

USUV strains were isolated from the brains of positive individuals using the C6/36 mosquito cell culture, as previously described [7]. Viral titres were determined by plaque assay as described previously [24] using A549 cell culture (human pulmonary epithelial cells, ATCC, CCL-185). The presence of viral antigen in the infected mosquito C6/36 and mammalian A549 cell lines was confirmed by flavivirus-specific immunofluorescence staining [7]. The cell infection rate was estimated in two independent experiments for each of the cell lines (1000 individual cells counted per cell line and replicate).

### Serological analyses

Serum samples taken from Mikado pheasants (*Syrmaticus mikado*) kept in a cage neighboring to infected Chilean tinamous in Brno Zoo and additional archived serum samples from several Czech zoological gardens kindly provided by Dr. Natalie Rudenko (Laboratory of Molecular Ecology of Vectors and Pathogens, Institute of Parasitology, Biology Centre CAS) (Supplementary Table 3) were screened for anti-flavivirus antibodies using ELISA (ID Screen Flavivirus Competition ELISA, Innovative Diagnostics, Grabels, France), following manufacturer’s instructions. Positive and borderline samples were subsequently tested using virus neutralization tests with USUV (strain 277TM10, lineage Europe 2; BCCO 50_0521), WNV (WNV EG-101) [25], and TBEV (European subtype strain Hypr; BCCO 50_0259) from the Biology Centre Collection of Organisms (www.bcco.cz) as described previously [26]. Briefly, the sera were inactivated for 30 min at 56 °C, diluted in a 96-well plate in cultivation media to reach the final dilution (including the volume of the added virus) of 25x, 50x, 100x, 200x, 400x, and 800x for USUV and TBEV and 40x, 80x, 160x, 320x, 640x, and 1280x for WNV. Individual viruses were added 50 PFU per well and incubated 90 min at 37 °C in 5% CO_2_. Then, 50,000 A549 cells per well were added and incubated for 5 days at 37 °C in 5% CO_2_. Subsequently, the plate was washed in sterile phosphate-buffered saline and stained in naphthalene blue. The neutralizing antibody titres were expressed as the reciprocal value of the serum dilution leading to 50% reduction in cytopathic effect (CPE) compared to controls. Sera reacting in dilutions > 40 were considered neutralizing. Four-fold differences between the cross-neutralizing antibody titres were considered proof of specific neutralizing capacity [27, 28].

## Results

### Pathological findings

In general, the birds in the zoological gardens exhibited common signs, such as weakness, tremor, and locomotion disorders. Neurological signs developed fully in last few days before death or euthanasia. Necropsy findings were significantly variable, even when comparing different individuals of the same species (*N. perdicaria*) ranging from slightly enlarged marbled spleen and/or liver, to pronounced pathological changes, including cachexy, hepato- and spleno-megaly with hyperaemia, haemorrhagic enteritis, pathological changes in the circulatory system (hypertrophied left ventricle, dilated cranial part of vena cava), slightly enlarged pancreas with miliary petechial haemorrhages. The severity of necropsy findings generally correlated with the duration of the symptoms. Detailed histopathological examination was done only in one Chilean tinamou and revealed small foci of perivascular lymphoplasmocytic encephalitis, lymphoplasmocytic hepatitis, focal cardiomyocyte dystrophy, pronounced pulmonary hyperaemia, and reactive hyperplasia of the lymphatic tissue of the spleen.

### Detection of USUV RNA in tissue samples

Altogether 22 individual birds were dissected, yielding 82 tissue samples. No WNV RNA was detected, whereas USUV RNA was found in 54.5% (*N* = 12) individuals and 45.1% (37) of the sampled tissues (Table 1). No statistically significant differences were observed in the number of viral RNA (vRNA) copies per gram of tissue when comparing species of birds or infected tissues (ANOVA). The average USUV target copy number was statistically significantly higher in captive birds (6.1E + 09 per g of tissue) than in free-living blackbirds (5.56E + 08 per g) but only in brain tissue comparison (t-test, *p* < 0.05). Differences between other tissues were not statistically significant (t-test).

**Table 1:** Bird tissues positive for USUV RNA as determined by RT-qPCR

Sample ID	Species	Positive tissues	Threshold cycle	Target copies/g of tissue
277TM	Common blackbird *(Turdus merula)*	brain	26.45	1.02E + 06
		coagulum (blood)	22.24	2.87E + 07^#^
		kidneys	20.18	1.05E + 08
		liver	21.19	4.97E + 07
		lungs	22.39	2.05E + 07
		spleen	20.12	1.10E + 08
278TM	Common blackbird *(Turdus merula)*	brain	14.67	1.58E + 09
		kidneys	17.29	7.85E + 08
		lungs	19.68	1.31E + 09
280TM	Common blackbird *(Turdus merula)*	brain	20.30	8.40E + 07
281NP	Chilean tinamou *(Nothoprocta perdicaria)*	brain	15.36	4.37E + 09
		heart	26.10	1.53E + 06
		kidneys	15.68	3.42E + 09
		liver	27.91	4.00E + 05
		lungs	22.82	1.74E + 07
		spleen	27.82	4.28E + 05
282NP	Chilean tinamou *(Nothoprocta perdicaria)*	brain	19.40	2.14E + 08
		coagulum (blood)	18.33	6.06E + 08^#^
		liver	17.83	6.97E + 08
		lungs	20.24	1.18E + 08
283NP	Chilean tinamou *(Nothoprocta perdicaria)*	brain	12.34	3.05E + 10
		liver	21.60	6.97E + 08
		lungs	30.84	4.62E + 04
284NP	Chilean tinamou *(Nothoprocta perdicaria)*	brain	16.73	1.58E + 09
		liver	15.32	4.48E + 09
286AF	Boreal owl *(Aegolius funereus)*	heart	28.78	2.12E + 05
292SP	Brahmini starling *(Sturnia pagodarum)*	brain	23.60	5.36E + 06
		cloacal swab	38.33*	-
		heart	26.17	9.08E + 05
		kidneys	27.86	4.17E + 05
		lungs	25.79	1.93E + 06
249GP	Eurasian pygmy owl *(Glaucidium passerinum)*	brain	24.63	1.71E + 06
		heart	37.46	3.44E + 02
		kidneys	27.53	5.32E + 05
		lungs	33.23	7.84E + 03
Cu700	Boreal owl *(Aegolius funereus)*	choanal/cloacal swabs	16.67	-
Cu701	Brahmini starling *(Sturnia pagodarum)*	choanal/cloacal swabs	27.77	-

* threshold cycle of the sample is out of the range of calibration curve, corresponding approximately to 4 copies per reaction; ^#^ copies/ml of blood or coagulum

### Plaque assay and virus isolation in cell cultures

Viral titres in RT-qPCR positive brain homogenates were determined using plaque assay reaching an average viral load of 3.9E + 06 PFU/g (Table 2). The paired t-test revealed statistically significant (approximately three logs) difference between the number of USUV target copies and replicating viral particles per g of tissue (*p* < 0.01).

**Table 2:** USUV RNA and viral loads per g of brain tissue in the wild and captive birds

Sample ID	Species	Wild/captive	Target copies/g of tissue	PFU/g of tissue
277TM	Common blackbird *(Turdus merula)*	wild	1.02E + 06	below detection limit
278TM	Common blackbird *(Turdus merula)*	wild	1.58E + 09	2.22E + 03
280TM	Common blackbird *(Turdus merula)*	wild	8.40E + 07	below detection limit
281NP	Chilean tinamou *(Nothoprocta perdicaria)*	captive	4.37E + 09	2.52E + 03
282NP	Chilean tinamou *(Nothoprocta perdicaria)*	captive	2.14E + 08	below detection limit
283NP	Chilean tinamou (*Nothoprocta perdicaria)*	captive	3.05E + 10	1.56E + 07
284NP	Chilean tinamou *(Nothoprocta perdicaria)*	captive	1.58E + 09	5.20E + 04
292SP	Brahmini starling *(Sturnia pagodarum)*	captive	5.36E + 06	below detection limit
249GP	Eurasian pygmy owl *(Glaucidium passerinum)*	captive	1.71E + 06	below detection limit

**Fig. 2:**
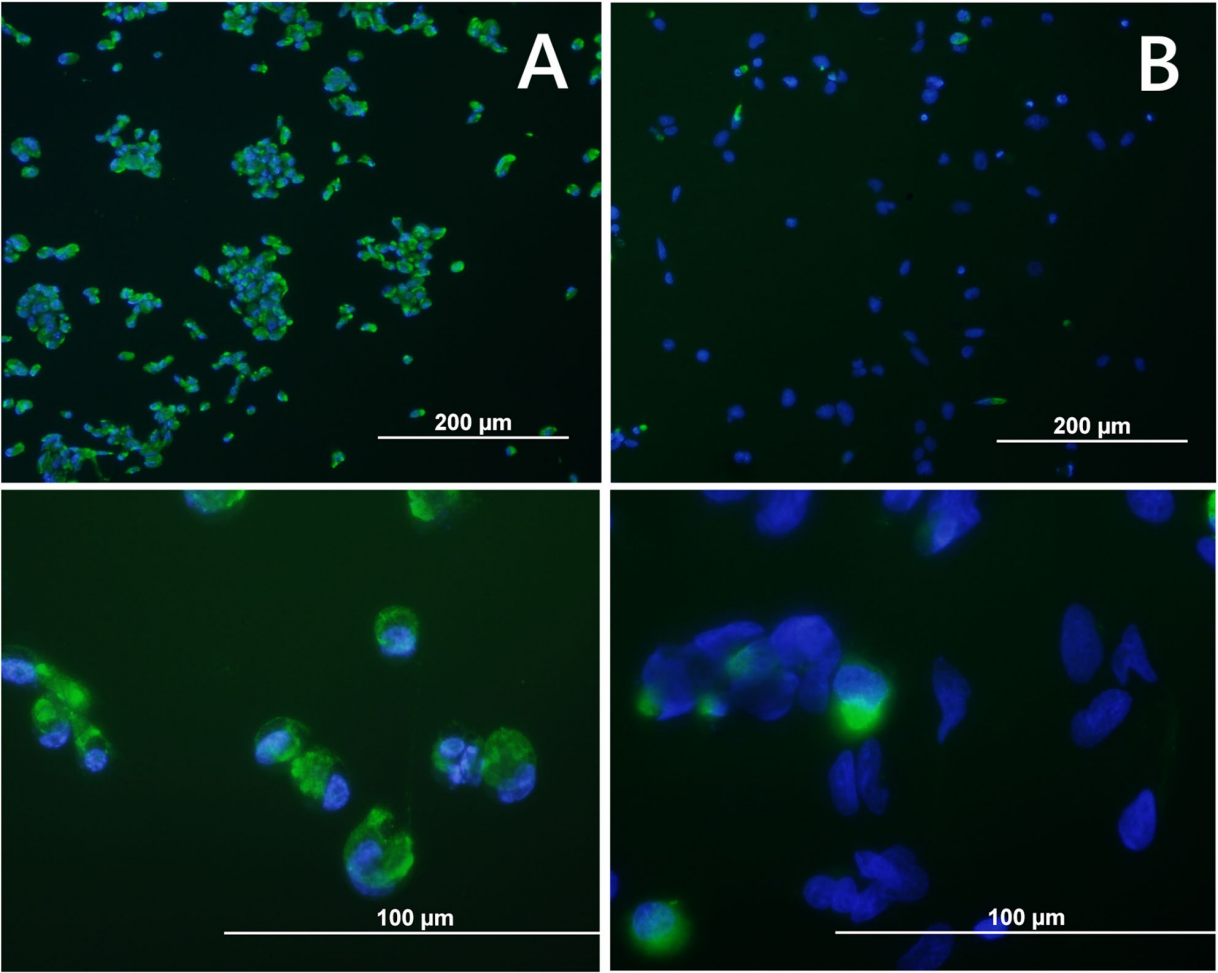
Indirect immunofluorescence staining of flaviviral E protein in mosquito and human cell cultures. Flaviviral E protein (green) in C6/36 mosquito cells (**a**) and A549 human pulmonary epithelial cells (**b**). Cell nuclei counterstained using DAPI (blue)

The virus isolation process was successful in eight out of nine USUV vRNA-positive brain homogenates using the C6/36 mosquito cells. Active virus propagation was confirmed using an A549 plaque assay, achieving average viral titres of 3.78E + 06 PFU/ml six days after infection, without any visible CPE in infected C6/36 cultures. The viral isolates are stored and available in the Biology Centre Collection of Organisms (www.bcco.cz). Immunofluorescence staining of flaviviral E protein revealed that in average 22% of the mammalian A549 cells were infected with the strain 277TM/10, while the C6/36 cells exhibited almost 100% infection rate (Fig. 2).

### Phylogenetic analyses

**Fig. 3:**
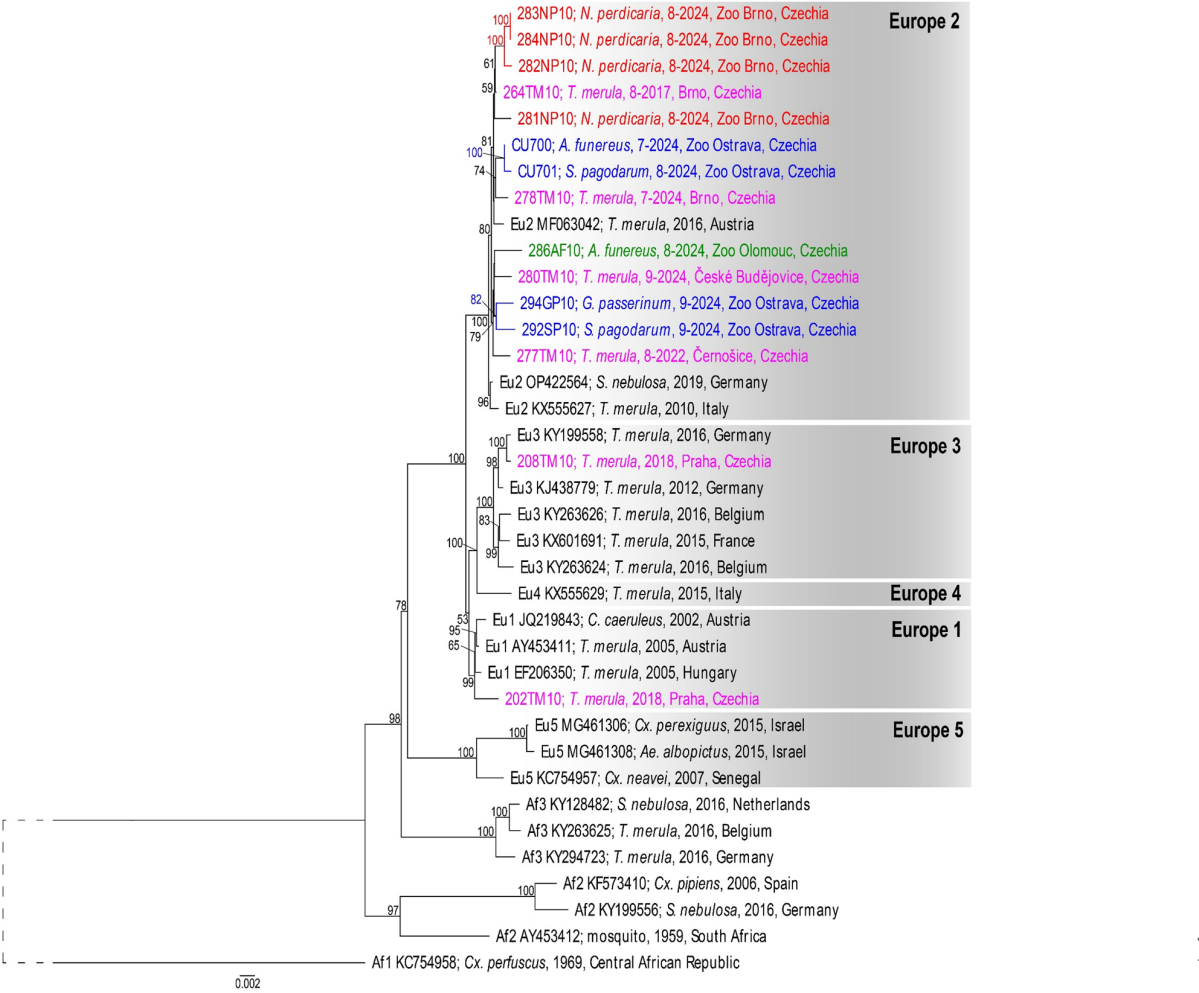
Maximum likelihood phylogenetic tree based near-complete genome (10,936 nt) nucleotide sequences of Usutu viruses. Sequences obtained in this study indicated by colour (red –Brno Zoo; blue –Ostrava Zoo; green –Olomouc Zoo; violet – free-living birds), sequences downloaded from the GenBank database in black. Tip labels include GenBank accession number/sample code, host species, date, and locality. Bootstrap supports are shown only for nodes with value > 50

**Fig. 4:**
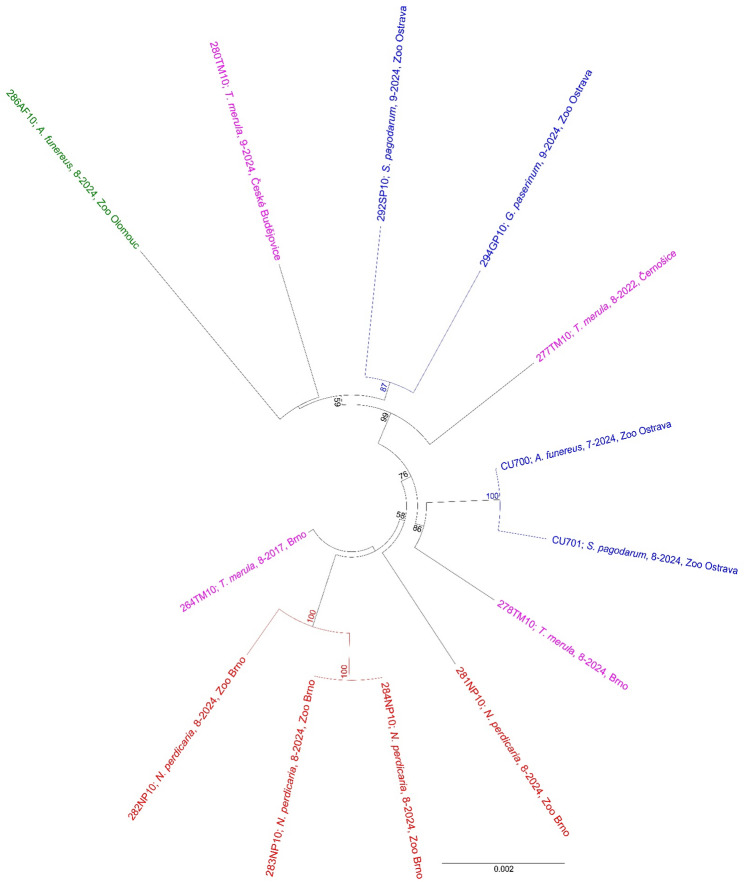
Unrooted maximum likelihood phylogenetic based on near-complete genome nucleotide sequences of Usutu viruses. Sequences obtained in this study indicated by colour (red –Brno Zoo; blue –Ostrava Zoo; green –Olomouc Zoo; violet – free-living birds). Bootstrap supports are shown only for nodes with value > 50

The nearly full-length genome nucleotide sequences (10,936 nt) were used to reconstruct phylogenetic relationships. Both the maximum likelihood (Figs. 3 and 4) and Bayesian inference (Supplementary Fig. 1, 2) approaches produced well-resolved phylogenetic trees with almost identical topologies. All USUV sequences obtained from dead birds in this study were assigned to the Europe 2 genetic lineage. In general, the sequences from zoo birds formed three geographically partially supported clusters. One cluster consists of sequences from birds in Brno Zoo (Figs. 3 and 4 in red) and an additional sequence from a free-living blackbird (violet) also found in Brno but in 2017. The second cluster contains sequences originating from two Ostrava Zoo birds (blue) that died 7–8/2024 and free-living blackbirds (violet) found in Brno in 2024, and Vienna (Austria) in 2016. The last cluster comprises the sole sequence obtained from Olomouc Zoo (green), the remaining two sequences from Ostrava Zoo (blue), from birds that died 9/2024, and two additional sequences from free-living blackbirds (violet) found in the western part of the country (approximately 280 km from Ostrava, 200 km from Olomouc) (Fig. 4).

**Fig. 5:**
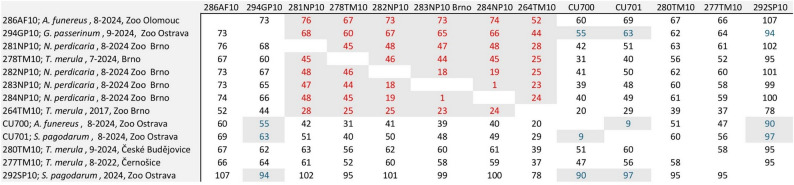
Pairwise nucleotide differences among the acquired near-complete genome sequences (10,396 nt) of Usutu viruses. The sequences from the Brno Zoo outbreak are in red, sequences from Ostrava Zoo outbreak are in blue

In the pairwise comparison the sequences from Brno Zoo differed in 1 (99.9% identity) to 48 nucleotide positions (99.5% identity). USUV sequences from the two Chilean tinamous (283NP10, 284NP10) were almost identical (a single nucleotide mismatch, 100% amino acid sequence identity), whereas 281NP10 differed significantly from all other sequences from Brno Zoo samples (47–48 nucleotide sequence differences, 8–9 amino acid differences). The differences among the nucleotide sequences originating from Ostrava Zoo samples ranged from 9 (99.9% identity) to 97 mismatches (99.1% identity) (Fig. 5). Notably the sequence of the sample 292SP10 contains a 37 nt deletion in the 3´UTR region compared to all other acquired sequences.

### Serological analyses

All samples positive or borderline in ELISA (Supplementary Table 3) were subjected to virus neutralization tests with the three flaviviruses that are known to occur in the area: USUV, WNV, and TBEV. Apart from a wolf serum with low titre of anti-USUV neutralization antibodies (NABs), all USUV or WNV neutralizing sera cross-reacted with both viruses (Table 3). No cross-neutralization was recorded for TBEV. Four-fold difference in NABs titre was reached only for a single serum of a Mikado pheasant housed in the cage neighboring with the Chilean tinamous at Brno Zoo allowing to conclude this serum as anti-USUV specific. Similarly, unequivocally anti-WNV NABs titres were found in a Wood owl (*Strix aluco*) from a private owner from the South Moravian region (close to Brno). The Wood owl had reported history of neurological disease (symptoms included torticollis, ataxia) and recovered fully. Additionally, one reindeer serum from Olomouc Zoo tested positive for anti-TBEV neutralizing antibodies.

**Table 3:** Titres of neutralizing antibodies against tick-borne encephalitis virus (TBEV), Usutu virus (USUV), and West nile virus (WNV)

Sample ID	Species	Origin	Year of sampling		Virus	
				TBEV	USUV	WNV
32	Gyrfalcon (*Falco rusticolus)*	private falconer	2024	< 25	50	160
36	Wood owl (*Strix aluco*)	private falconer	2024	< 25	*50*	**1280**
N3	Reindeer (*Rangifer tarandus*)	Olomouc Zoo	prior to 2014	**100**	< 25	< 40
N42	Wolf (*Canis lupus*)	Plzeň Zoo	prior to 2014	< 25	**50**	< 40
230	Mikado pheasant (*Syrmaticus mikado*)	Brno Zoo	2024	< 25	400	160
226	Mikado pheasant (*Syrmaticus mikado*)	Brno Zoo	2024	< 25	200	80
218	Mikado pheasant (*Syrmaticus mikado*)	Brno Zoo	2024	< 25	400	160
223	Mikado pheasant (*Syrmaticus mikado*)	Brno Zoo	2024	< 25	**> 800**	*160*

The titres were determined in previously ELISA-positive or borderline sera using virus neutralization tests. The titres are expressed as the reciprocal value of the serum dilution causing a 50% reduction in cytopathic effect. Four-fold and higher difference in neutralization titre or > 40 combined with negative results for the remaining results was considered a threshold to assign the response to one of the viruses. Titres of neutralizing antibodies are indicated in bold and cross-neutralizing titres in italics

## Discussion

Zoological gardens have unique conditions conducive to virus transmission [29, 30]. Many bird species from different parts of the world with different susceptibility to the infection are caged in proximity of each other, making the transmission of the infection easier and potentially more stable [30]. The application of preventive measures targeting the presence of vectors is limited in the zoos, due to requirements for open enclosures and freely accessible water reservoirs. Vaccination is complicated due to the variable immune responses of different species, lack of approved bird- and virus-specific vaccines, and significant costs associated with vaccinating large numbers of individuals [13, 14, 31]. Consequently, captive bird populations are at high risk of USUV infections, which are frequently associated with substantial losses.

USUV infections have been reported in numerous zoological gardens across Europe, including neighboring Austria and Germany [11, 12, 32–34]. Our study was initially prompted by several fatal cases of USUV infection in Chilean tinamous at the Brno zoological garden, which devastated the local breeding population and significantly impacted the European/global zoo breeding program. Of the seven individuals housed together, five succumbed to the infection (one individual was not necropsied). These are the first reported cases of USUV infection in this species worldwide. Serological evidence of exposure was reported only in distantly related greater rheas (*Rhea americana*), emus (*Dromaius novaehollandiae*) [33, 35], and an ostrich (*Struthio camelus*) [32]. Also, in the case of Brahminy starlings there are no previous reports of USUV infection in this species. On the other hand, passerine birds as well as owls including Boreal owl and a Eurasian pygmy owl are well known to be sensitive to flavivirus infections [11]. In the zoo settings, substantial diversity has been observed among exposed species, ranging from commonly infected individuals of Strigiformes [11, 12, 32] to isolated cases of less expected species, such as Humboldt penguins, Egyptian vultures or ratites mentioned above [32, 33, 35].

In sensitive species of birds, USUV infection is usually multisystemic, targeting the central nervous system and internal organs. Hepatomegaly and splenomegaly are considered major macroscopic pathological findings associated with the infection [11, 36, 37], which is consistent with our findings. USUV RNA has previously been detected in various internal organs, most frequently in the brain [7, 9, 37, 38]. In the present study, the brain was confirmed as the most suitable tissue for the USUV RNA screening, as detectable vRNA was found in brains of all USUV-positive birds, whereas different other tissues tested negative in some individuals. The amount of viral RNA per gram of brain tissue was in average 3 logs higher than the amount of replicating virus particles as determined by plaque assay. A similar ratio was reported previously for other mosquito-borne flaviviruses [39, 40]. The difference might be explained by generation of defective viral particles, release of free viral RNA from lysed cells, and other processes in the flaviviral replication cycle [41]. Brain tissues of captive USUV-positive birds contained statistically significantly higher vRNA copy numbers compared to brain tissues of free-living birds. One possible explanation is, that captive birds are more likely to be found sooner after death and their cadavers transferred to freezers preventing RNA degradation. Nevertheless, there are several important limitations possibly affecting this finding, such as limited number of individuals and comparing several different species of captive birds with blackbirds which is the only free-living bird species positive in our study. USUV RNA was found also in cloacal/choanal swabs of one Boreal owl and one Brahminy starling, and in a cloacal swab of another Brahminy starling. The possibility of non-vector mediated transmission has been suggested previously based on USUV shedding in oral, pharyngeal and cloacal secretions [42–47], along with evidence for such transmission in WNV [48]. In both Brno Zoo and Ostrava Zoo, we found USUV infected birds housed either together or in close vicinity of each other. Nevertheless, we are unable to conclude on the route of infection.

In the Czech Republic, USUV has been previously detected in blackbirds [1, 7], mosquitoes [7, 49], and as an autochthonous human infection [16]. Although the Europe 1, 3, and Africa 3 genetic lineages were detected in blackbirds and mosquitoes in the same (South Moravia) region previously [1, 7, 49], phylogenetic analyses assigned the sequences of all the newly detected viruses in our study to the Europe 2 lineage. The relatively low variability of detected lineages may be due to the limited number of samples analyzed or to the specific conditions of zoological gardens. Furthermore, the Europe 2 lineage appears to currently dominate in Europe [50]. Notably, it was also reported to be more virulent in a mice model compared to Africa 3 and Europe 3 [51]. This lineage is also frequently associated with cases of human neurological infections, while Europe 3 and Africa 3 have been found in healthy donors [3, 15, 52]. Although all four Chilean tinamous were housed in the same enclosure and appeared to have been infected around the same time, in one individual a significantly genetically different USUV strain was detected. These findings could indicate either rapid intra-host evolution of the virus [53–55] or co-circulation of two different strains in Brno Zoo. We assume the latter possibility is more likely, given that the divergent sequence from the tinamou was more similar to a sequence obtained from a free-living blackbird found in the same city in 2017 than to those from the other three tinamous.

As shown previously using local strains of Europe 1 and 3 lineages [7], also in the case of our Europe 2 isolate, the mosquito C6/36 cells were nearly 100% infected but exhibited no CPE, whereas the mammalian cells were infected in a lower proportion and the infection was associated with CPE. These differences might be at least partly associated with the differences in immune response. While the mosquito cell line is deficient in the RNA interference pathway [56], in the mammalian A549 cells USUV replication might be considerably reduced due to interferon response [57].

Although blood from the Chilean tinamous was unavailable for testing, several serum samples from birds or other animals from zoological gardens, as well as all four serum samples obtained from Mikado pheasants that were caged directly next to the USUV-positive Chilean tinamous in Brno Zoo tested positive for anti-USUV/anti-WNV antibodies. One of the Mikado pheasants had anti-USUV antibody titres more than four times higher than its anti-WNV NAB titres, which is considered proof of anti-USUV antibody specificity [27, 28]. Due to the low probability of a previous WNV infection, it is likely that the other three pheasants also had specific antibodies to USUV rather than to WNV, but did not reach the threshold difference between the NABs titres. Also, we could not determine in this study whether the antibody response resulted from a recent or previous infection, as the class of antibodies was not identified. Of the historical serum samples from zoological gardens, one wolf (*C. lupus*) serum sample from Plzeň Zoo was found weakly anti-USUV-positive, which would indicate the presence of the virus in the western part of the country as early as 2013. Additionally, a single TBEV-neutralizing serum sample was confirmed from a reindeer (*R. tarandus)* from Olomouc Zoo, as reported previously by Širmarová et al. [26]. Interestingly, anti-WNV NABs were found in the serum sample of a wood owl from a private owner from the South Moravian region, where WNV circulation has been confirmed [16, 49, 58, 59]. Cross-neutralization was observed among USUV- and WNV-antibody-positive sera (Table 3), which was demonstrated also previously [32, 33, 35]. Although no approved avian vaccine is currently available for flavivirus infections, several studies have reported the use of equine anti-WNV vaccines providing a certain level of protection in specific bird species [13, 14]. The cross-reactivity described above might thus indicate possible cross-protectivity to USUV [46], although further studies are needed to confirm the efficiency [60].

## Conclusions

We have described cases of fatal USUV infections in free-living and captive birds in the Czech Republic. Chilean tinamous were found highly sensitive to the virus as five of seven individuals housed together succumbed to infection. USUV-positive birds were found in three different zoological gardens in the eastern part of the country, in proximity to areas with known endemic circulation of USUV and WNV [9, 12, 16, 49, 58, 59, 61]. However, USUV-infected blackbirds and mosquitoes have also been detected in the southwestern and central regions [7], indicating that the virus is widely distributed across the Czech Republic.

Both USUV and WNV are capable of causing fatal disease in captive endangered, rarely bred, or otherwise valuable avian species. Given the limited preventive options, particularly in zoological gardens, rapid identification of infections, and prompt isolation of susceptible species represent an effective mitigation strategy. Furthermore, as both viruses have the potential to be transmitted to humans [15, 16, 61], surveillance data from birds are an important contribution to public health monitoring and early warning systems for human disease in the One Health context [30].

## Supplementary Information

Below is the link to the electronic supplementary material.

Supplementary Material 1

## Data Availability

The datasets supporting the conclusions of this article are included within the article and its additional files. The acquired nucleotide sequences were submitted to the GenBank database under accession numbers PX210786-PX210800.
