# Supplementary material for: First report of Usutu virus fatal infections in Chilean tinamous (Nothoprocta perdicaria), brahminy starlings (Sturnia pagodarum), and multiple other bird species in zoological gardens and wildlife in the Czech Republic
Source: One Health Outlook. 2026 Jan 2;8:4. doi: 10.1186/s42522-025-00191-3 (PMC12828963; doi:10.1186/s42522-025-00191-3)
Supplement: Supplementary file 1 — Supplementary Material 1 [file 42522_2025_191_MOESM1_ESM.docx]

Supplementary Table 1: List of birds tested for the presence of Usutu and West Nile virus RNA

| **Sample code** | **Species** | **Locality** | **Date of death/**  **finding** | **Wild or captive** | **Sampled tissues** |
| --- | --- | --- | --- | --- | --- |
| 277TM | *Turdus merula* | Černošice | 8/2022 | wild | blood/coagulum, brain, kidneys, lung, liver, spleen |
| 278TM | *Turdus merula* | Brno | 7/2024 | wild | brain, kidneys, lung, liver, spleen |
| 279TM | *Turdus merula* | Mokré | 7/2024 | wild | brain |
| 280TM | *Turdus merula* | České Budějovice | 9/2024 | wild | brain |
| 281NP | *Nothoprocta perdicaria* | Brno Zoo | 18/8/2024 | captive | brain, heart, kidneys, lungs, liver, spleen |
| 282NP | *Nothoprocta perdicaria* | Brno Zoo | 17/8/2024 | captive | blood/coagulum, brain, lungs, liver |
| 283NP | *Nothoprocta perdicaria* | Brno Zoo | 25/8/2024 | captive | brain, lungs, liver |
| 284NP | *Nothoprocta perdicaria* | Brno Zoo | 21/8/2024 | captive | brain, liver |
| 285 | *Alectroenas madagascariensis* | Plzeň Zoo | 6/9/2024 | captive | brain, liver |
| 286AF | *Aegolius funereus* | Olomouc Zoo | 20/8/2024 | captive | heart, liver |
| 287BB | *Bubo bubo* | Kroměříž | 4/2024 | captive | spleen |
| 288PF | *Poicephalus flavifrons* | Prostějov | 8/2024 | captive | brain |
| 289PP | *Perdix perdix* | Vyškov | 8/2024 | captive | liver |
| 290EoR | *Eolophus roseicapilla* | Břeclav | 7/2024 | captive | multiple internal organs |
| 291ER | *Erithacus rubecula* | Brno | 9/2024 | wild | brain |
| 292SP | *Sturnia pagodarum* | Ostrava Zoo | 6/9/2024 | captive | brain, cloacal swab, choanal swab, heart, kidneys, lungs, liver |
| 293SS | *Sturnia sinensis* | Ostrava Zoo | 10/9/2024 | captive | brain, heart, kidney, liver, spleen |
| 294GP | *Glaucidium passerinum* | Ostrava Zoo | 8/9/2024 | captive | brain, heart, kidney, lungs, liver, spleen |
| 295FF | *Falco femoralis​* | Hodonín | 9/2024 | captive | brain, kidney, lungs, spleen |
| 296FF | *Falco femoralis​* | Hodonín | 9/2024 | captive | brain, kidneys, lungs |
| CU700 | *Aegolius funereus* | Ostrava Zoo | 29/7/2024 | captive | choanal/cloacal swabs |
| CU701 | *Sturnia pagodarum* | Ostrava Zoo | 26/8/2024 | captive | choanal/cloacal swabs |

Supplementary Table 2. Sequences of primer and probes used for multiplex one-step RT-qPCR detection of Usutu and West Nile virus RNA in the bird samples.

| **Target** | **Primer/probe** | **Sequence** | **Reference** |
| --- | --- | --- | --- |
| Usutu virus | UsuFP | CAAAGCTGGACAGACATCCCTTAC | Nikolay et al. 2014 |
|  | UsuP | FAM-AAGACATATGGTGTGGAAGCCTGATAGGCA-BHQ |  |
|  | UsuRP | CGTAGATGTTTTCAGCCCACGT |  |
| West Nile virus | INEID F1 | AGTAGTTCGCCTGTGTGAGC | Eiden et al. 2010 |
|  | INEID probe | CY5-AATCCTCACAAACACTACTAAGTTTGTCA-BHQ2 |  |
|  | INEID R1 | GCCCTCCTGGTTTCTTAGA |  |

Supplementary Table 3. Serum samples from zoos and private owners used for the detection of anti-flaviviral antibodies.

| **Sample ID** | **Species** | **Origin** | **ELISA** |
| --- | --- | --- | --- |
| 218 | Mikado pheasant (*Syrmaticus mikado*) | Brno Zoo | **positive** |
| 223 | Mikado pheasant (*Syrmaticus mikado*) | Brno Zoo | **positive** |
| 226 | Mikado pheasant (*Syrmaticus mikado*) | Brno Zoo | **borderline** |
| 230 | Mikado pheasant (*Syrmaticus mikado*) | Brno Zoo | **positive** |
| 1 | Galah (*Eolophus roseicapilla*) | private owner | **negative** |
| 2 | Grey parrot (*Psittacus erithacus*) | private owner | **negative** |
| 3 | Burrowing parrot (*Cyanoliseus patagonus*) | private owner | **negative** |
| 4 | Grey parrot (*Psittacus erithacus*) | private owner | **negative** |
| 5 | Senegal parrot (*Poicephalus senegalus*) | private owner | **negative** |
| 6 | Blue-fronted amazon (*Amazona aestiva*) | private owner | **negative** |
| 7 | Red-spectacled amazon (*Amazona pretrei*) | private owner | **negative** |
| 8 | Grey parrot (*Psittacus erithacus*) | private owner | **negative** |
| 9 | Blue-fronted amazon (*Amazona aestiva*) | private owner | **negative** |
| 10 | Blue-fronted amazon (*Amazona aestiva*) | private owner | **negative** |
| 11 | Grey parrot (*Psittacus erithacus*) | private owner | **negative** |
| 12 | Grey parrot (*Psittacus erithacus*) | private owner | **negative** |
| 13 | Blue-and-yellow macaw (*Ara ararauna*) | private owner | **negative** |
| 14 | Black-headed parrot (*Pionites melanocephalus)* | private owner | **negative** |
| 15 | Black-headed parrot (*Pionites melanocephalus)* | private owner | **negative** |
| 16 | Moluccan eclectus (*Eclectus roratus*) | private owner | **negative** |
| 17 | Yellow-crowned amazon (*Amazona ochrocephala*) | private owner | **negative** |
| 18 | White cockatoo (*Cacatua alba*) | private owner | **negative** |
| 19 | Senegal parrot (*Poicephalus senegalus*) | private owner | **negative** |
| 20 | Hyacinth macaw (*Anodorhynchus hyacinthinus*) | private owner | **negative** |
| 21 | Grey parrot (*Psittacus erithacus*) | private owner | **negative** |
| 22 | Grey parrot (*Psittacus erithacus*) | private owner | **negative** |
| 23 | Grey parrot (*Psittacus erithacus*) | private owner | **negative** |
| 24 | Blue-and-yellow macaw (*Ara ararauna*) | private owner | **negative** |
| 25 | Grey parrot (*Psittacus erithacus*) | private owner | **negative** |
| 26 | Blue-fronted amazon (*Amazona aestiva*) | private owner | **negative** |
| 27 | Blue-winged macaw (*Primolius maracana*) | private owner | **negative** |
| 28 | Grey parrot (*Psittacus erithacus*) | private owner | **negative** |
| 29 | Grey parrot (*Psittacus erithacus*) | private owner | **negative** |
| 30 | Grey parrot (*Psittacus erithacus*) | private owner | **negative** |
| 31 | Grey parrot (*Psittacus erithacus*) | private owner | **negative** |
| 32 | Gyrfalcon (*Falco rusticolus*) | private owner | **positive** |
| 33 | Senegal parrot (*Poicephalus senegalus*) | private owner | **negative** |
| 34 | Blue-and-yellow macaw (*Ara ararauna*) | private owner | **negative** |
| 35 | Grey parrot (*Psittacus erithacus*) | private owner | **negative** |
| 36 | Tawny owl (*Strix aluco*) | private owner | **positive** |
| N1H | Snow leopard (*Panthera uncia*) | Olomouc Zoo | **negative** |
| N2H | Lar gibon (*Hylobates lar)* | Plzeň Zoo | **negative** |
| N3H | African buffalo (*Syncerus caffer*) | Dvůr Králové nad Labem Zoo | **negative** |
| N4H | Common blackbird (*Turdus merula*) | Hluboká nad Vltavou Zoo | **negative** |
| N2 | Cheetah (*Acinonyx jubatus*) | Olomouc Zoo | **negative** |
| N3 | Reindeer (*Rangifer tarandus*) | Olomouc Zoo | **borderline** |
| N5 | Leopard (*Panthera pardus*) | Olomouc Zoo | **negative** |
| N6 | Elk (*Alces alces*) | Olomouc Zoo | **negative** |
| N27 | Red lechwe (*Kobus leche*) | Plzeň Zoo | **negative** |
| N28 | Wapiti (*Cervus canadensis*) | Plzeň Zoo | **negative** |
| N29 | Mountain goat (*Oreamnos americanus*) | Plzeň Zoo | **negative** |
| N30 | Mountain goat (*Oreamnos americanus*) | Plzeň Zoo | **negative** |
| N31 | Mountain goat (*Oreamnos americanus*) | Plzeň Zoo | **negative** |
| N36 | Blesbok (*Damaliscus pygargus phillipsi*) | Plzeň Zoo | **negative** |
| N39 | Mountain goat (*Oreamnos americanus*) | Plzeň Zoo | **negative** |
| N40 | Black-backed jackal (*Lupulella mesomelas*) | Plzeň Zoo | **negative** |
| N41 | Wolf (*Canis lupus*) | Plzeň Zoo | **negative** |
| N42 | Wolf (*Canis lupus*) | Plzeň Zoo | **borderline** |
| N43 | Wolf (*Canis lupus*) | Plzeň Zoo | **negative** |


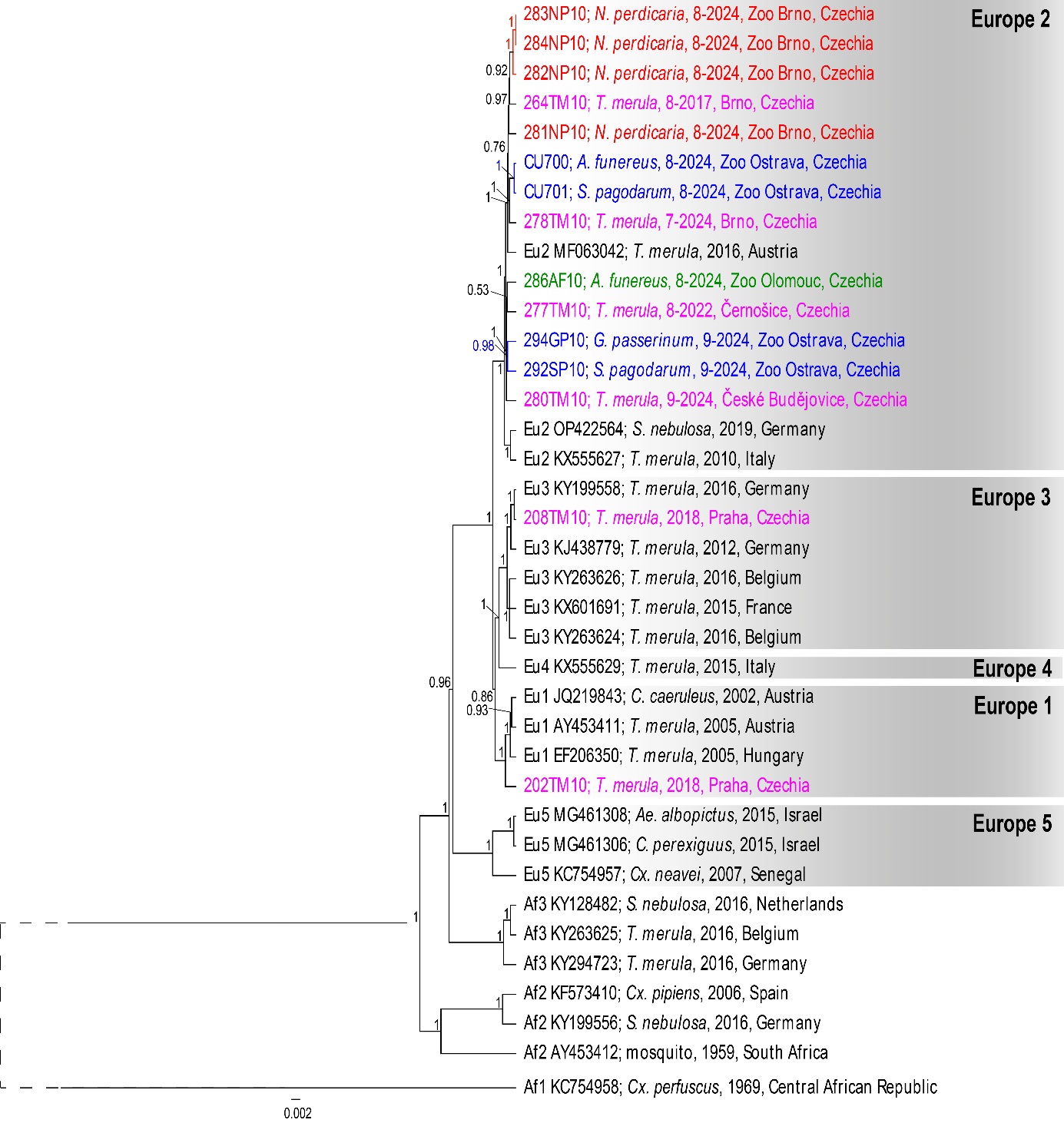


Supplementary Figure 1: Bayesian inference phylogenetic trees based on nearly whole genome (10,936 nt) nucleotide sequences of Usutu virus. Sequences obtained in this study are indicated by colour (red –Brno Zoo; blue–Ostrava Zoo; green –Olomouc Zoo; violet – free-living birds), sequences downloaded from the GenBank database are in black. Tip labels include GenBank accession number/sample code, locality, date and host species. Bootstrap supports are shown only for nodes with value <0.5.

**
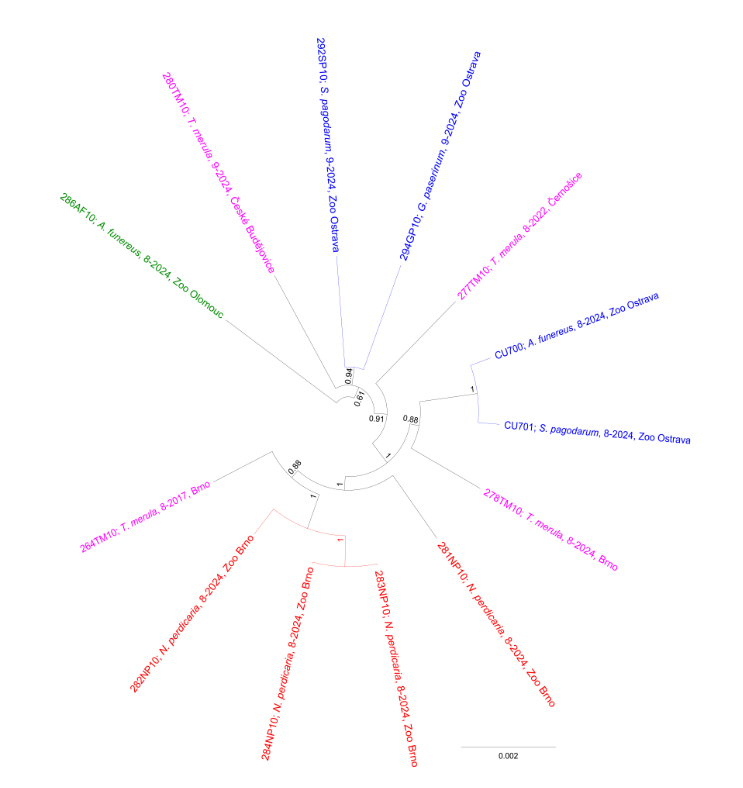
**Supplementary Figure 2. Unrooted Bayesian inference phylogenetic based on nearly whole genome nucleotide sequences of Usutu virus sequences from this study; red –Brno Zoo; blue –Ostrava Zoo; green –Olomouc Zoo; violet – free-living birds). Bootstrap supports are shown only for nodes with value <50.
